# Retraction Note: A paradigm shift in cell-free approach: the emerging role of MSCs-derived exosomes in regenerative medicine

**DOI:** 10.1186/s12967-025-06275-y

**Published:** 2025-03-04

**Authors:** Soudeh Moghadasi, Marischa Elveny, Heshu Sulaiman Rahman, Wanich Suksatan, Abduladheem Turki Jalil, Walid Kamal Abdelbasset, Alexei Valerievich Yumashev, Siavash Shariatzadeh, Roza Motavalli, Farahnaz Behzad, Faroogh Marofi, Ali Hassanzadeh, Yashwant Pathak, Mostafa Jarahian

**Affiliations:** 1 Department of Biochemistry and Molecular Biology, School of Medicine, Shahid Sadoughi University of Medical Sciences, Yazd, Iran; 2 DS & CI Research Group, Universitas Sumatera Utara, Medan, Indonesia; 3 College of Medicine, University of Sulaimani, Sulaymaniyah, Iraq; 4 Department of Medical Laboratory Sciences, Komar University of Science and Technology, Sulaymaniyah, Iraq; 5 Faculty of Nursing, HRH Princess Chulabhorn College of Medical Science, Chulabhorn Royal Academy, Bangkok, 10210 Thailand; 6 Faculty of Biology and Ecology, Yanka Kupala State University of Grodno, Grodno, Belarus; 7 Department of Health and Rehabilitation Sciences, College of Applied Medical Sciences, Prince Sattam Bin Abdulaziz University, Al Kharj, Saudi Arabia; 8 Department of Physical Therapy, Kasr Al-Aini Hospital, Cairo University, Giza, Egypt; 9 Sechenov First Moscow State Medical University, Moscow, Russia; 10 Department of Pharmacology, School of Medicine, Shahid Beheshti University of Medical Sciences, Tehran, Iran; 11 Stem Cell Research Center, Tabriz University of Medical Sciences, Tabriz, Iran; 12 Research Institute of Bioscience and Biotechnology, University of Tabriz, Tabriz, Iran; 13 Department of Hematology, Faculty of Medicine, Tabriz University of Medical Sciences, Tabriz, Iran; 14 Department of Applied Cell Sciences, School of Advanced Technologies in Medicine, Tehran University of Medical Sciences, Tehran, Iran; 15 Taneja College of Pharmacy, University of South Florida, Tampa Florida, USA; 16 German Cancer Research Center, Toxicology and Chemotherapy Unit (G401), 69120 Heidelberg, Germany

**Retraction Note: Journal of Translational Medicine (2021) 19:302**

10.1186/s12967-021-02980-6

The Editor in Chief has retracted this article. An investigation by the Publisher has found a number of articles, including this one, which share similar concerns, involving but not limited to, irregularities with respect to submission and authorship. The Editor-in-Chief therefore no longer has confidence in the results and conclusions presented in this article. Abduladheem Turki Jalil, Siavash Shariatzadeh, Roza Motavalli, Faroogh Marofi, Yashwant Pathak and Mostafa Jarahian disagree to this retraction. Soudeh Moghadasi, Marischa Elveny, Heshu Sulaiman Rahman, Wanich Suksatan, Walid Kamal Abdelbasset, Alexei Valerievich Yumashev, Farahnaz Behzad and Ali Hassanzadeh did not respond to correspondence from the Editor about this retraction.

